# Analyzing Procedural Time and Its Relationship to Performance in Microsurgical Training: A Pilot Study

**DOI:** 10.7759/cureus.90516

**Published:** 2025-08-19

**Authors:** Pablo J Villanueva, Taku Sugiyama, Yelena Akelina, Hector I Rodriguez, Francisco Salguero

**Affiliations:** 1 Microsurgical Laboratory, University of Buenos Aires, Buenos Aires, ARG; 2 Neurosurgery, Hokkaido University, Sapporo, JPN; 3 Simulation, Columbia University Vagelos College of Physicians and Surgeons, New York, USA; 4 Engineering, National University of Salta, Salta, ARG; 5 Informatics, Universidad Nacional de Cuyo, Mendoza, ARG

**Keywords:** computer platform for surgical evaluation, microsurgery simulator, microsurgical training, super microsurgery, surgical skill assessment

## Abstract

Introduction

Objective measures and precise variable selection are cornerstones of skill assessment. While the quantity and quality of movements in microsurgery are evaluated using advanced technology and innovative methods, it remains a challenge to move beyond the notion that skill improvement is synonymous with faster procedures. A critical analysis reveals that quality does not always align with speed, nor does improved performance necessarily mean faster performance. This study aims to analyze and describe the progression of time variables during skill training and to provide new tools for their thorough assessment and evaluation.

Methods

This trial followed a published microsurgical protocol measuring time/errors at defined stages. Procedures were chronologically recorded, capturing per-session counts, inter-session intervals, and protocol data. Misat-APP® collected data, exported to PostgreSQL®, and analyzed via Python® scripts. Samples were categorized by mistake levels to better evaluate temporal progression patterns. Data dispersion was analyzed by the coefficient of determination (R²) for regression modeling. A standard deviation method was applied (±2 SD) to refine the sample, standardize calculations, and eliminate outliers often found when working with sensitive variables. After the first analysis, the target was aimed exclusively at mistake-free procedures to establish the average-zero (AVO) index. This benchmark was then cross-referenced with qualitative error markers, revealing a consistent mistake-free speed range named "Safe Pace" (±0.2 SD from the AVO). Statistical significance was set at p < 0.05 throughout all analyses.

Results

The trial included 158 procedures performed over nine months and 75 sessions. After removing procedures with major mistakes, the sample accounted for 150 procedures. The total microscope time was 61 hours, 22 minutes, and 55 seconds. The time to complete the task (TCT) presented significant fluctuations (slowest = 3,057 seconds at the fourth procedure; fastest = 890 seconds at the 81st procedure), tracing a decreasing pattern with a significant dispersion level (R² = 0.601). While observing inter-session time gaps, and after refining the sample by removing outliers (±2 SD), a strong correlation was evidenced between gaps longer than 10 days and their impact on the TCT at the next session (paired T-test, p = 0.009). When calculating the AVO index (1,463.27 seconds), it was cross-referenced with the full sample, and the widest and significant "Safe Pace" corresponded to ± 0.2 SD (1,362.88-1,563.67 seconds; p = 0.012). Other findings suggested that time is also linked to the number of procedures in the same session, procedural strategy (magnification level, instruments, sequence of steps), final end-product quality, and the operator's attitude.

Conclusion

Time-related performance metrics were analyzed throughout the trial. Findings suggest that time alone is a low-reliability indicator for skill assessment, showing high sensitivity to external factors. The concept of an average time and a speed range where procedural errors tend to decrease ("Safe Pace") may offer a useful benchmark for both advising operators during training and evaluating their performance. These findings, while consistent with prior studies, require further investigation for confirmation. Additionally, data from experienced operators indicated that excessively fast procedures could be associated with a higher risk of errors.

## Introduction

Objective measurements and the selection of key variables are crucial in skill assessment [1,2]. The time taken and performance speed are essential benchmarks for evaluating an operator's proficiency in any field [3]. While faster procedures might seem advantageous, they are not necessarily better. Performance must balance speed with the rigor of achieving accurate and high-quality results. The time taken for an operator's movements to complete a task serves as a quantitative measure, while the criteria for task completion define the qualitative assessment.

Movement quantity can be assessed by the time to complete a task (TCT), which reflects not only the operator’s hand speed but also their motion economy, naturally linked to skill level and expertise. TCT values are sensitive but are influenced by many factors, thus lacking specificity. The quality of the procedure can be graded by the final product's appearance or the number of mistakes made during the task, which should be objectively scored by experts or through another validated methodology. Multiple studies have investigated the quantity-quality phenomenon using various tools, techniques, and technologies, each with distinct cost, complexity, and accuracy profiles [4-9].

The speed of an operator is a valuable feature, but it should not overshadow other crucial aspects, such as precision, consistency, and overall effectiveness. Achieving a balance ensures comprehensive performance improvement without compromising essential qualities. A thorough investigation of time-related variables offers a promising foundation for advancing the understanding of hand-skill assessment. Within the microsurgical context, this evaluation gains an additional dimension because surgical strategy and technique appear to significantly influence procedural outcomes, thereby underscoring the relevance of time as a focal point of study.

The primary objective of this pilot study was to analyze and describe the behavior of the time variable within a comprehensive and controlled microsurgical trial. This in-depth examination provided the basis for developing new conceptual instruments - such as performance indices and calculation models - aimed at improving the assessment of surgical skills in training environments.

## Materials and methods

Training protocol

This pilot trial employed a validated microsurgical training protocol designed to objectively evaluate the acquisition of hand skills. This protocol was chosen because it offers clearly defined, progressive microsurgical tasks using the human placenta as a realistic simulator for end-to-end vessel anastomosis. A detailed description of the protocol and its associated learning curve analysis can be found in previous publications [10,11]. Additionally, to minimize bias from biological variability, we incorporated a difficulty grading scale specifically for placenta-simulator quality standardization [12].

Time variables and procedural mistakes

The records evaluated included two time variables: TCT and inter-session time (IST). When multiple procedures were performed within the same session, the time between them was considered to be zero, as the next procedure began immediately after the previous one concluded.

To objectively assess procedural mistakes, the previous protocol error-scoring system was applied:

Minor mistake (1 point): A technical error that did not compromise the final outcome and was potentially and easily repairable (e.g., minor leakage during patency testing).

Major mistake (5 points): A critical error that prevented the successful completion of the procedure or required substantial corrective action (e.g., an arterial wall tear during dissection).

To improve the precision of TCT values and their interpretation, this mistake scoring system served two purposes:

Refining the sample for a more accurate observation of time-related variables (TCT, IST): Because major mistakes disproportionately increase procedural time and distort temporal analyses, we excluded all procedures containing major errors at this specific stage of analysis. This resulted in a sample that included only procedures with a mistake score of four points or less.

Cross-referencing TCT with mistake levels: This approach allowed us to identify interesting situations where significant changes in TCT values were accompanied by corresponding variations in the mistake score. This analysis enabled the calculation of the average time of a sample composed only of flawless procedures - the AVO index - and the identification of a related speed range where procedures remained without major mistakes (named “safe pace”).

The AVO index was defined as the mean TCT value of all procedures with zero mistakes. Then, by using the AVO as a benchmark, three speed ranges were defined:

Safe pace: The central range, defined as being within ± 0.2 standard deviations (SD) from AVO. This specific parameter was chosen because it provided the broadest range that remained completely free of major mistakes and showed optimal statistical significance (details in the Results section).

Faster pace: All values that were lower than (AVO - 0.2 SD).

Slower pace: All values that were higher than (AVO + 0.2 SD).

Data processing

**Figure 1:**
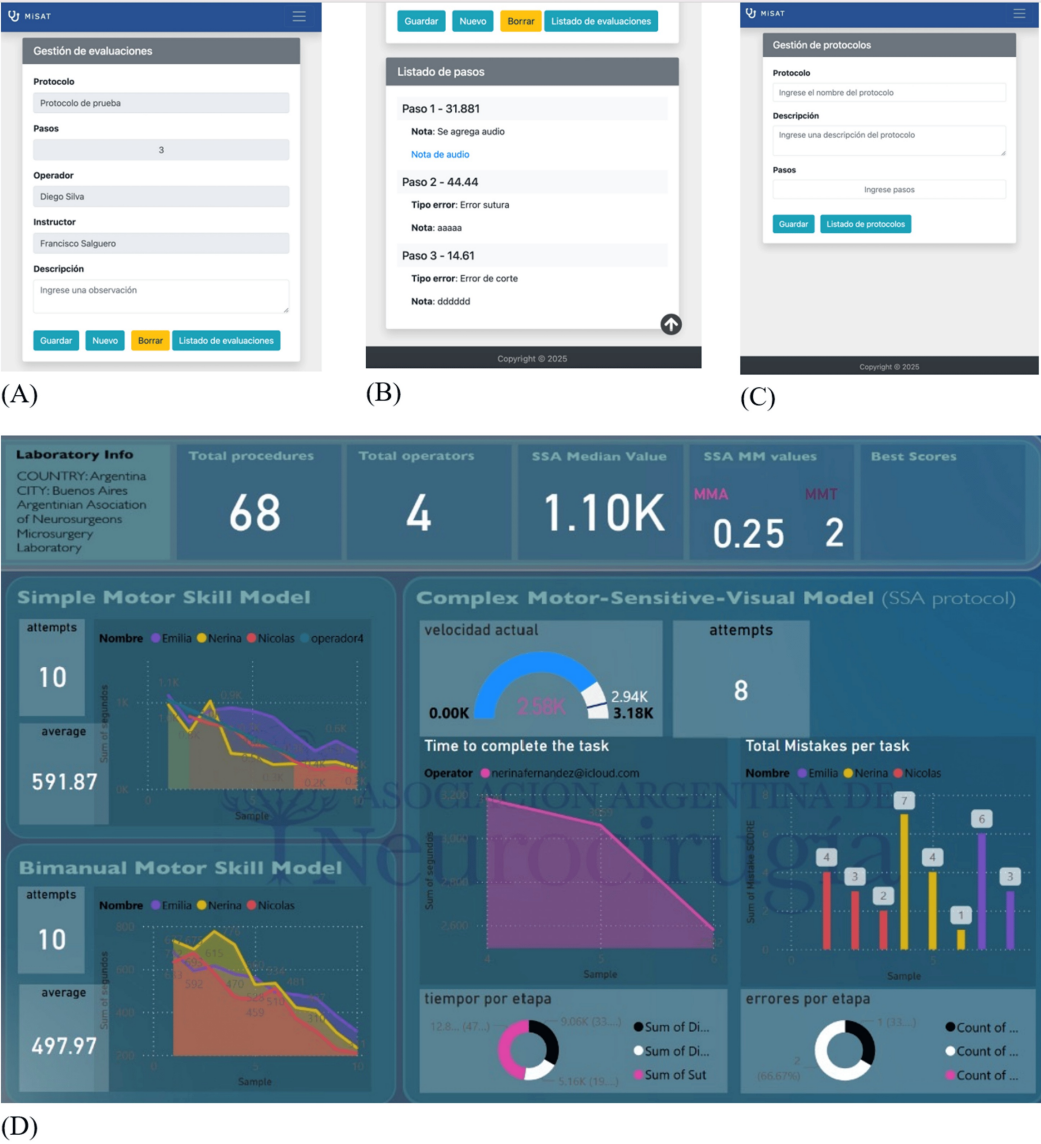
Cellphone-based application for microsurgical data recording. (A) Main screen to select the working protocol and user details. (B) Results of a just-ended session (this information, once the procedure ended, is automatically and immediately recorded at the main dataset, MySQL® and Postgres® compatible). (C) Screenshot of protocol selection, instructions, and options. (D) Automatically and live dashboard for all the operators during a session. The number of operators, sessions, and dates can be selected to evaluate different stages of a trainee cohort or similar segregation methods. Source: Authors' own elaboration MiSAT-App ® screenshots and Microsoft PowerBI ® dashboard.

**Figure 2:**
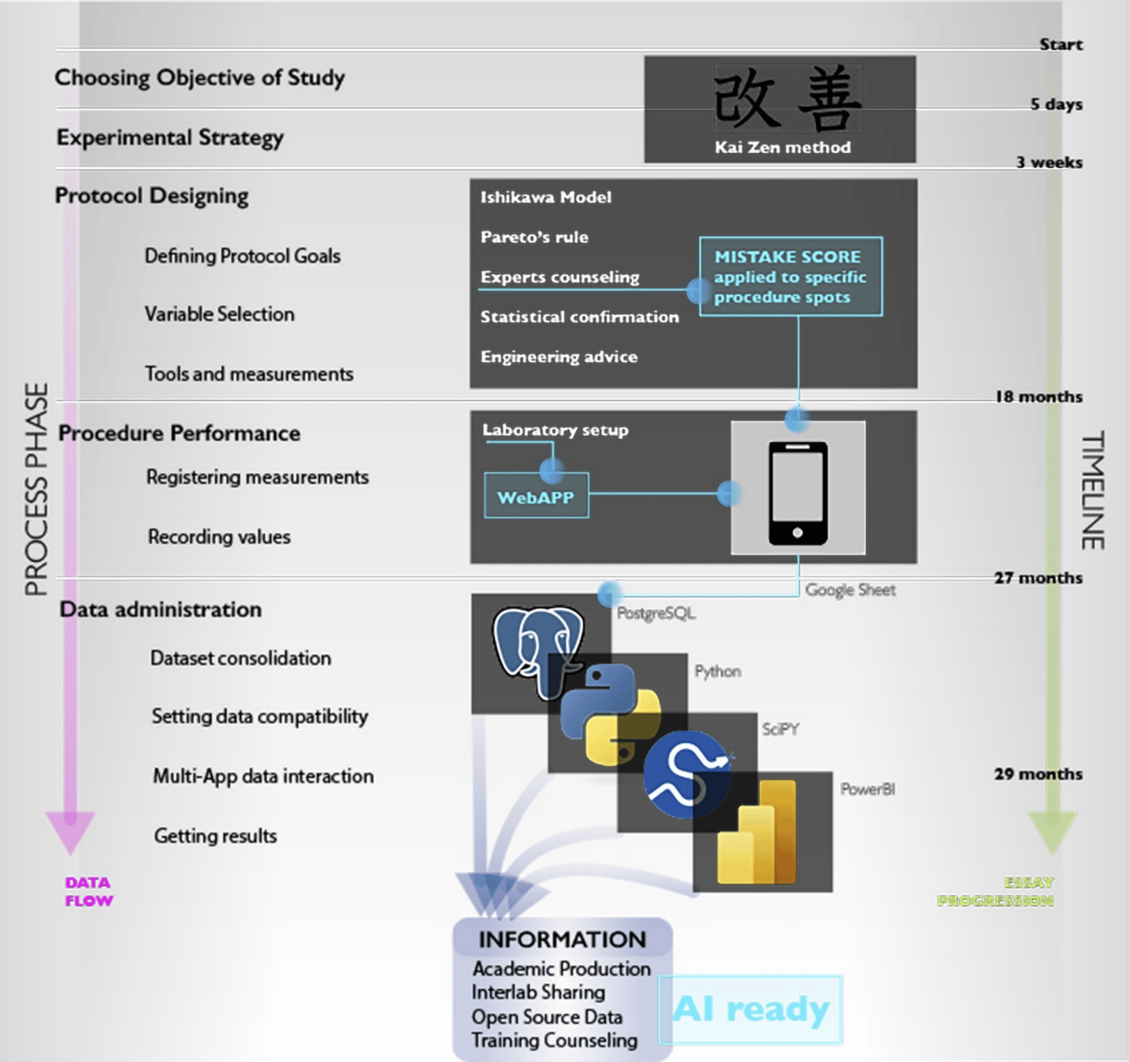
Data flow and data processing diagram. This infographic illustrates the data flow from the initial requirement (microsurgical training) to the final goal (multi-purpose information), depicted by phases and time consumed at each phase. These phases were scientifically based, and evidence was generated (training protocols, index and values of interpretation) when not available. The entire system has been verified to be reliable across various scenarios and also being ready for data intelligence algorithms. Source: Author's own elaboration

All sample records were registered using MisatAPP® (powered by CDLAB, Argentina), a dedicated web application that allowed the operator to upload required data from his procedures (improving data collection and reducing bias), access his own live-cast results, and receive performance-related reports (screenshots in Figure 1). Once the data were generated and uploaded, it was automatically anonymized, validated, and exported to a formal PostgreSQL® database. This setup allowed for flexible and reliable interaction (by using Python® scripts) between variables and dedicated statistical software and prepared the scenario for engaging with artificial intelligence. The main structure of the raw data can be evaluated in Table 1, and a graphic description of data processing can be found in Figure 2.

**Table 1:** Raw data records (sample of 13 procedures). Sample of 13 procedures data frame, generated by Misat-APP. Variables for mistakes and times were individualized by stages (stage 1 “360”, stage 2 “adventitia”, and stage 3 “suture”). A new variable was calculated (from the procedures date values, to measure (in days) the time gap between sessions). The complete raw table can be found in the appendix section. Source: Authors' own elaboration

Procedure	Date	Mistakes				Time				
		360	Adventitia	Suture	Total	360	Adventitia	Suture	Total	Gap
1	24-04-18			1	1	776	203	1159	2138	0
2	24-04-19		1	1	2	1002	859	752	2613	1
3	24-04-19				0	408	562	901	1871	0
4	24-04-20		1		0	484	648	1925	3057	1
5	24-04-24				0	619	452	1865	2936	4
6	24-04-25			1	1	690	646	1458	2794	1
7	24-04-25			1	1	404	397	840	1641	0
8	24-04-26			2	1	435	135	1028	1598	1
9	24-04-26				0	446	946	690	2082	0
10	24-04-26				0	519	593	822	1934	0
11	24-04-29				1	246	505	1188	1939	3
12	24-04-30				0	472	508	962	1942	1
13	24-05-02		1	1	2	711	648	1079	2438	2

A single operator, trained in microsurgical techniques and protocol-specific procedures, performed all trial procedures. While this single-operator design was deemed adequate for the current study, future dataset consolidation could be strengthened by incorporating records from multiple operators.

Statistical analysis

For overall data: simple counting and a logarithmic trend line. Data dispersion was calculated as the coefficient of determination (R²).

For multiple sessions and inter-session analysis: when multiple procedures were conducted within a single session, the TCT of the first and second procedures were compared using a paired T-test (when more than two procedures were found in the same session, no significant difference was found for procedures following the second one). When the IST was 10 days or longer (this gap was found to be of optimal statistical significance), the difference between the TCT before and after this interval was analyzed. Outliers were identified (±2 SD) and removed to improve precision. The correlation between this difference and the IST value was analyzed using a paired T-test.

For the AVO index: simple average (mean value) and standard deviation for further investigation. Statistical significance was set at a p-value < 0.05 for all evaluated procedures.

## Results

Overall data and learning curve

**Figure 3:**
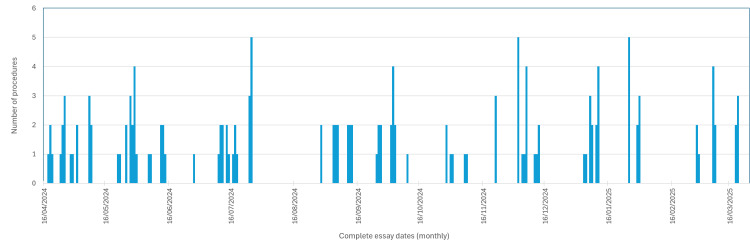
Training sessions and their number of procedures during the complete trial (chronological order). The horizontal axis represents the complete time of the trial (11 months), with marks at every month. The vertical axis shows the number of procedures performed at each session. Each bar represents a trial session, and its height correlates with the number of procedures performed at that particular session. Blank spaces between bars represent time gaps between sessions (days without any microsurgical activity). Source: Author's own elaboration

This trial included 158 procedures (eight were excluded for having a major mistake). The study was performed over eleven months in 76 sessions, with an average of 1.97 procedures per session (Figure 3).

**Figure 4:**
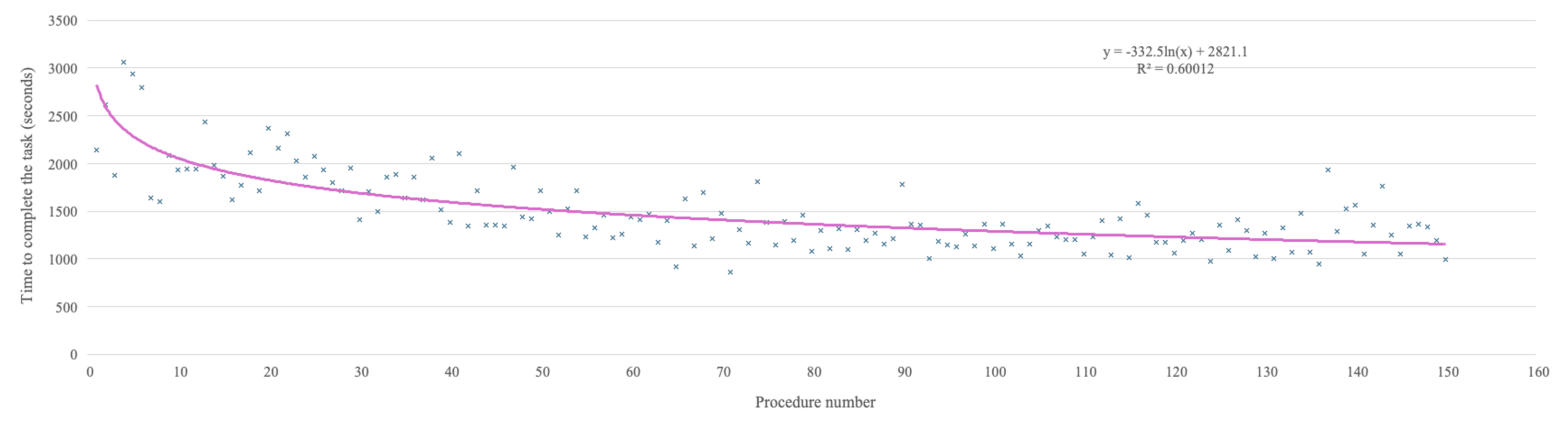
Scatter plot of time-to-complete task (TCT) across trial procedures. The horizontal axis displays the consecutive procedure number (1–150), while the vertical axis represents TCT (seconds). Individual records (blue dots) exhibit high dispersion (R² = 0.601). The pink trend line confirms a significant downward learning curve, aligning with prior studies [10,11]. Source: Authors' own ellaboration

The total microscope time is 61 hours, 21 minutes, 51 seconds. The TCT presented a wide range of values (slower one = 3,057 seconds at the fourth procedure; faster one = 890 seconds, 81st procedure), tracing a decreasing trend line (Figure 4) with a significant dispersion level (R^2^ = 0.6001).

Multiple procedures within a single session

**Figure 5:**
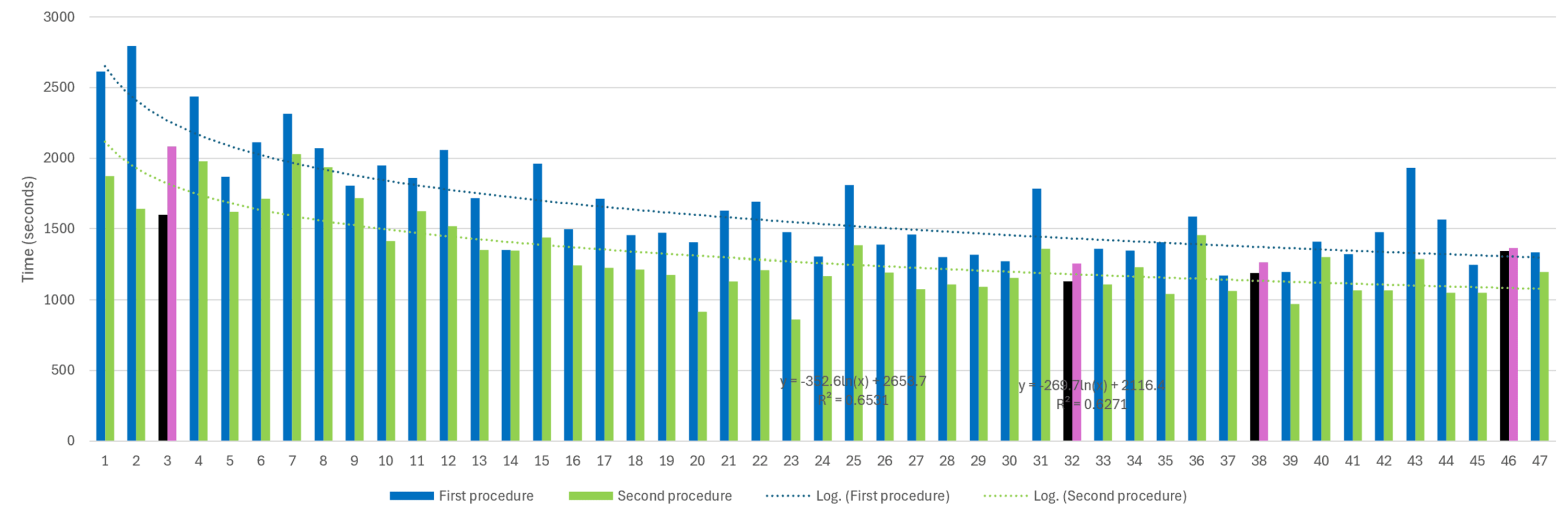
Comparison of time-to-complete task (TCT) between first and second procedures within the same session. The horizontal axis shows consecutive sessions accounting more than one procedure (47 sessions). The vertical axis represents time (in seconds). Vertical bars are presented in pairs at each session, with its height representing the TCT value. When the first procedure was slower than the second one, colors are blue and green, respectively (common pattern, 93,62%). When the pattern inverted, the firs procedure is shown in black and the second in pink color. Trend lines from both remained significantly apart, even at plateau levels (T-test p < 0.00001). Source: Authors' own elaboration

Data was segregated by "sessions accounting for more than one procedure," and 47 sessions met this criterion. After segregation, the TCT values of procedures within the same session were compared. In 44 out of 47 sessions (93.62%), the second procedure was significantly faster than the first (Figure 5). The average time of the first and second procedures was 1,690.23 seconds and 1,313.83 seconds, respectively (paired T-test, p < 0.00001).

IST and microsurgical performance

**Figure 6:**
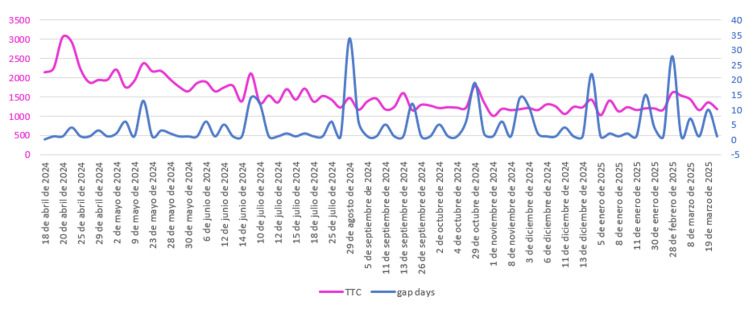
Temporal association between inter-session intervals and procedural speed: time-to-complete task (TCT) and inter-session time (IST) correlation. The horizontal axis represents the time along the trial. The vertical axis represents the TCT in seconds (pink-colored labels at the left and the primary line) and the IST in days (blue-colored labels at the right and the secondary line). Correlation between TCT (pink line) and IST (blue line) across the trial duration exhibited aligned peaks, suggesting longer gaps between sessions correspond to increased procedural times. Source: Author's own ellaboration

A correlation was found between microsurgical performance and a significant IST. The first procedure performed after a long IST showed significantly higher TCT levels (paired T-test, p < 0.009). This correlation was evidenced by analyzing TCT variations in relation to IST levels (Figure 6).

**Figure 7:**
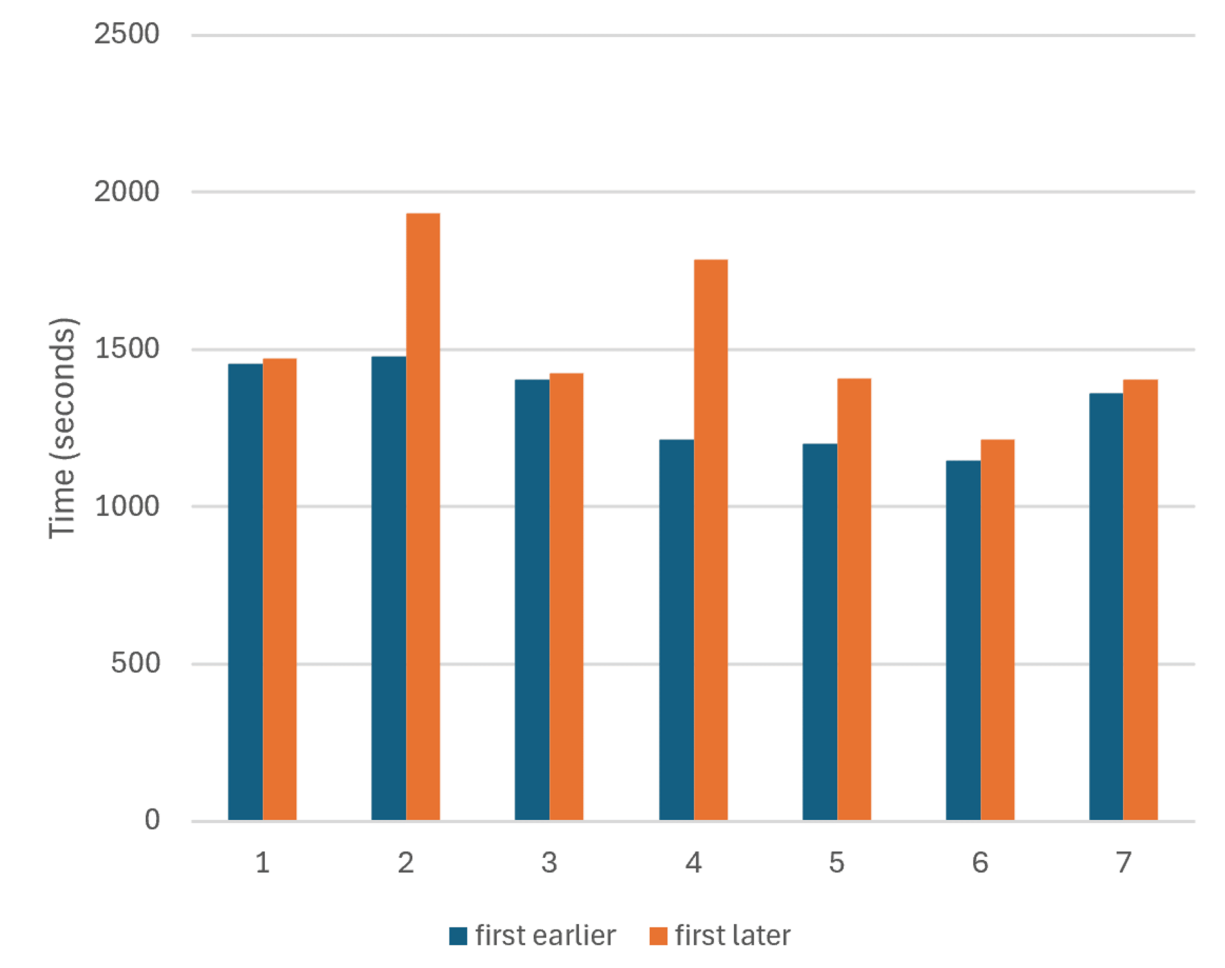
Impact of prolonged inter-session intervals (>10 days) on the procedural speed: a paired comparison of time-to-complete task (TCT). The horizontal axis represents the session pairs separated by ≥10 days, with blue bars indicating the first procedure of the earlier session and orange bars the first procedure of the later session. The vertical axis displays time (seconds). Seven session pairs met inclusion criteria (outliers beyond ± 2 SD were excluded). Bar heights correspond to time-to-complete task (TCT) values. The observed difference was statistically significant (paired T-test, p = 0.009). Source: Authors' own elaboration

During this trial, an IST greater than 10 days occurred on eight occasions. For each of these pairs, TCT levels were compared between the first procedure of the earlier session and the first procedure of the later session (Figure 7). After this segregation, the results were refined by calculating the standard deviation of the sample and removing outliers, which were defined as a value falling outside the range of ± 2 SD from the mean (sample mean = 1,451.81, sample SD = 360.99).

AVO index

Our results identified a descending trend in TCT that corresponded closely with a decrease in the number of mistakes, both of which are indicators of skill acquisition. Interestingly, after reaching a plateau in the learning curve, major mistake rates showed a slight rise for faster procedures.

To better understand this situation, a thorough cross-reference between TCT and mistake levels was performed on the complete dataset. As mentioned in the methods section, this analysis included registries for all mistake levels. The results of this examination allowed us to identify a speed range where the mistake level was close to zero. This benchmark (the “average-zero,” defined in the Methods section) was used to calculate the widest range found to remain error-free (± 0.2 SD of the AVO; sample mean = 1,463 seconds; range from 1,362.88 to 1,563.67 seconds). This range (termed the “safe pace”) represented the procedure speed of the sample that was free of major mistakes (p = 0.012).

An additional interpretation showed that deviating from this "safe pace" in either direction carried a higher risk of major mistakes. Specifically, a slower pace accounted for an 11.67% major mistake risk, while a faster pace accounted for a 1.37% major mistake risk (Table 2).

**Table 2:** Segregation of procedures using the average-zero index to define a significant central range (safe pace). This table presents data stratified according to the average-zero (AVO; see Methods) benchmark. The central category was defined as values within ±0.2 SD of AVO (TCT range: 1,389.94-1,536.61 seconds), representing the widest range with optimal statistical significance (p = 0.012). The analysis incorporated the complete dataset (158 registries), including procedures with major mistakes, to enable comprehensive cross-referencing. Source: Authors' own elaboration

Pace Category	Number of Procedures	Number of Major Mistakes	Percentage of Major Mistakes
Faster (<1389.94 s)	73	1	1.37%
Safe Pace (1389.94-1536.61 s)	25	0	0.00%
Slower (>1536.61 s)	60	7	11.67%

## Discussion

Continuous improvement-oriented analysis of a procedure integrates surgical, technical, and engineering domains [13]. Identifying critical elements within a procedure is essential for its favorable evolution. These elements become "improvement opportunities." Once identified, prioritizing them based on impact enables effective monitoring of significant variables. This approach initiates an improvement cycle, wherein regular, incremental adjustments lead to substantial positive changes. Several authors emphasize the importance of expert opinion in determining precisely what to observe and where to focus [14,15].

In manual procedures, monitoring requires a comprehensive understanding, study, and observation of both the quantity and quality of hand movements [16-18]. While movement quantity can be approximated by the TCT, this metric is often crude and imprecise [16], showing wide variability due to subtle, frequently overlooked differences. This variability is especially pronounced in microsurgery, making it a critical area for analyzing time-related data, including TCT, inter-procedure intervals, performance order, and gap-time of inactivity between sessions.

Overall data

Figure 3 summarizes the trial framework. The X-axis represents chronological order with regular monthly intervals. The bars indicate sessions, with height reflecting the number of procedures per session. The blank gaps between bars denote operator inactivity. TCT correlated with skill acquisition (lower TCT indicating faster procedures). However, further steps were necessary to verify this relationship, such as correlating TCT with error rates and final product quality. This suggests that, while TCT is a sensitive variable, it is also context-dependent. Figure 4 shows a decreasing trend line (skill correlation) with high dispersion (R² = 0.601), indicating that TCT should not be used as the sole marker of skill progression. These data correlated very well with prior studies [10,11].

Multiple sessions and inter-session

Further analysis revealed 47 sessions with more than one procedure. In 44 of these (93.62%), the first procedure was significantly slower than the second (p < 0.00001). Figure 5 illustrates these sessions, with separate lines for the TCT values of the first and second procedures. This suggested that the initial procedure may serve as a "warming-up" phase, potentially improving hand precision and fluidity, possibly due to increased relaxation, confidence, or familiarity with the simulator tissue response.

IST, measured in days without microsurgical activity, was also incorporated to interpret performance. Figure 6 shows IST peaks corresponding to longer inactivity periods. When TCT values are plotted chronologically alongside IST, both display synchronized peaks, demonstrating that prolonged gaps in practice negatively affect performance, resulting in longer task completion times. This relationship was statistically confirmed (paired T-test, p = 0.009) for IST periods exceeding 10 days (Figure 7). It was also observed that, after returning to the laboratory following a long IST, the operator’s skill appeared to normalize after one procedure (typically 18-25 minutes). These findings support the recommendation for surgeons to avoid extended breaks and maintain a regular, even minimal, training routine. The effectiveness of this named routine should be properly examined.

Cross-reference between time and mistakes variables (average-zero index)

This study aimed to thoroughly examine how procedural speed affects outcomes. To refine this analysis, time data were cross-referenced with other variables, combining quantitative (TCT) and qualitative (major mistakes) evaluations. After a first data analysis, to determine whether a specific pace or rhythm yields safer or more effective results, the new AVO index was calculated and then used to identify a speed range associated with zero major mistakes.

As seen in Table 2, this analysis defined three speed zones: the safe pace zone (±0.2 SD around AVO), the faster zone (below the regular zone, with a low mistake risk), and the slower zone (above the regular zone, with a high mistake risk). SD was particularly useful for precisely defining the central range in an easily reproducible manner, while its validity was also straightforward to demonstrate. In this trial, the case was at ±0.2 SD, where TCT values accounted for no major mistakes, the speed range was as wide as possible, and validation yielded better scores (p = 0.012).

SD thresholds improved dataset representativeness and statistical reliability while providing a natural framework for the interaction of quantitative and qualitative variables. From this point, several interpretations emerged: (a) if a safe pace exists, it should be calculated individually for each operator and procedure; (b) slower procedures may reflect underlying difficulties or the occurrence of error-prone conditions during execution; (c) a slower pace might also indicate a lack of skill or prolonged inactive time (long IST) without training; and (d) the slight increase in risk observed in faster procedures could correlate with factors such as overconfidence, insufficient precaution, or an undue emphasis on speed.

Regardless of the cause, the concept of a "safe pace" offers an interesting point of view for guidance, teaching, and identifying productive training rhythms. It also encourages deliberate execution of procedures, avoiding unnecessary haste, in pursuit of safe and high-quality outcomes. While the findings in Table 2 require further validation, they open new avenues for exploring how time-related variables (time, speed, pace) can inform microsurgical skill assessments and procedural evaluations. They also highlight the need for careful interpretation of these metrics.

It is also worth noting that the operator reported that varying magnification levels and modifying instruments or strategies can influence TCT, reinforcing the idea that TCT is affected by multiple external factors. These observations, along with time-related data, also aligned with previous studies [10,11], particularly regarding the low confidence in TCT as an independent skill marker. Overall, the trial provided a comprehensive view of time-related variables, offering valuable insights for skill assessment.

Limitations

Additional statistical tests, a larger series, and a multi-operator framework (aiming to enhance generalizability, vary operator experience, and include feedback surveys) are needed to confirm the findings of this study.

Future directions

Several instances for further investigation emerged during this trial and its interpretation. The data collected are promising but require validation through more complex methodologies, such as multi-operator frameworks and ANOVA testing. The AVO index presents an opportunity to study its behavior along the learning curve. Its use - either as a standalone metric or to define a safe procedural pace - raises questions about its value, range, and interpretive meaning. This need for new data positions the current research as a foundation for future studies.

## Conclusions

Time-related variables were found to be sensitive but lacked specificity as an independent skill marker. This assertion is supported by the high dispersion levels of TCT (R² = 0.6001), even after refining the sample by removing outliers. The TCT also showed significant interaction with external variables (e.g., IST influence (p < 0.009); the order within a session (p < 0.00001); and other annotations from the operator). Interestingly, the TCT (while being cross-referenced with other qualitative variables) demonstrated the ability to identify different paces of performance, showing a correlation with quality and safety levels (p < 0.012).

The observations throughout this trial allowed for a thorough description of the evolution of time-related variables, providing various insights and nuances relevant to skill training and proper assessment interpretations.

## Appendices

**Table 3:** Complete dataset. The complete trial records are presented in this table. Source: Authors' own elaboration

Procedure	Date	Mistakes				Time				Gap
		360	Adventitia	Suture	Total	360	Adventitia	Suture	Total	
1	April 18, 2024			1	1	776	203	1159	2138	0
2	April 19, 2024		1	1	2	1002	859	752	2613	1
3	April 19, 2024				0	408	562	901	1871	0
4	April 20, 2024		1		0	484	648	1925	3057	1
5	April 24, 2024				0	619	452	1865	2936	4
6	April 24, 2024				5	498	756	1923	3177	
7	April 25, 2024			1	1	690	646	1458	2794	1
8	April 25, 2024			1	1	404	397	840	1641	0
9	April 26, 2024			2	1	435	135	1028	1598	1
10	April 26, 2024				0	446	946	690	2082	0
11	April 26, 2024				0	519	593	822	1934	0
12	April 29, 2024				1	246	505	1188	1939	3
13	April 30, 2024				5	346	646	801	1793	
14	April 30, 2024				5	511	429	813	1753	
15	April 30, 2024				0	472	508	962	1942	1
16	May 2, 2024		1	1	2	711	648	1079	2438	2
17	May 2, 2024		1		1	366	573	1039	1978	0
18	May 8, 2024				6	474	599	1223	2296	6
19	May 8, 2024		1		1	444	338	989	1619	0
20	May 8, 2024			1	1	292	473	646	1767	0
21	May 8, 2024				0	648	308	1321	2113	1
22	May 9, 2024			1	1	484	273	957	1713	0
23	May 9, 2024				0	483	423	1473	2373	13
24	May 22, 2024			2	2	477	460	1320	2164	1
25	May 23, 2024			2	2	384	548	1478	2314	3
26	May 26, 2024				0	288	746	856	2028	0
27	May 26, 2024				0	426	194	947	1853	2
28	May 28, 2024			1	1	712	131	1150	2072	0
29	May 28, 2024			1	1	791	240	1216	1936	0
30	May 28, 2024				0	480	302	988	1804	1
31	May 29, 2024			1	1	514	458	776	1715	0
32	May 29, 2024			1	1	481	496	1200	1949	1
33	May 30, 2024			1	1	253	340	651	1413	0
34	May 30, 2024		1	1	2	422	387	1055	1702	0
35	May 30, 2024			1	1	260	170	1127	1500	0
36	May 30, 2024			1	1	203	242	869	1856	1
37	May 30, 2024				5	473	392	1003	1868	
38	May 31, 2024			1	1	745	619	838	1889	6
39	June 6, 2024			1	1	432	256	1086	1640	1
40	June 7, 2024				0	298	365	1037	1860	5
41	June 12, 2024			1	1	458	379	942	1624	0
42	June 12, 2024			1	1	303	620	948	2057	1
43	June 13, 2024			1	1	489	240	956	1517	0
44	June 13, 2024				0	321	282	797	1378	1
45	June 14, 2024			1	1	299	405	1332	2107	14
46	June 28, 2024			1	2	370	115	927	1343	12
47	July 10, 2024				5	473	190	1343	2006	
48	July 10, 2024			1	2	301	284	1009	1715	1
49	July 11, 2024			1	1	422	183	971	1350	0
50	July 11, 2024				0	196	194	777	1351	1
51	July 12, 2024				0	380	277	769	1347	0
52	July 12, 2024				0	301	506	1097	1959	2
53	July 14, 2024				0	356	279	876	1440	0
54	July 14, 2024				0	285	376	643	1423	1
55	July 15, 2024				0	404	213	1249	1715	2
56	July 17, 2024			1	1	253	375	793	1496	1
57	July 18, 2024			1	1	328	167	798	1246	0
58	July 18, 2024				0	281	416	761	1520	1
59	July 19, 2024				0	343	150	1082	1713	6
60	July 25, 2024			1	1	481	256	606	1228	0
61	July 25, 2024				1	366	223	825	1326	0
62	July 25, 2024				0	278	253	935	1454	1
63	July 26, 2024			1	1	266	298	688	1217	0
64	July 26, 2024				0	231	411	630	1261	0
65	July 26, 2024			1	1	220	277	932	1435	0
66	July 26, 2024			1	1	226	549	677	1415	0
67	July 26, 2024				0	189	264	676	1470	34
68	August 29, 2024				0	530	370	582	1176	0
69	August 29, 2024				0	224	385	692	1403	6
70	September 4, 2024			2	2	326	138	573	915	0
71	September 4, 2024				0	204	442	882	1626	1
72	September 5, 2024				0	302	225	637	1132	0
73	September 5, 2024			1	1	270	376	1010	1692	1
74	September 6, 2024			1	1	306	238	773	1211	0
75	September 6, 2024				0	200	440	740	1478	5
76	September 11, 2024				0	298	159	498	860	0
77	September 11, 2024				1	203	378	614	1302	1
78	September 12, 2024			1	1	310	331	594	1168	0
79	September 12, 2024				0	243	572	995	1810	1
80	September 13, 2024				0	243	238	845	1384	0
81	September 13, 2024				0	301	192	824	1147	12
82	September 25, 2024				5	230	289	1091	1610	
83	September 25, 2024				0	131	278	885	1388	1
84	September 26, 2024			1	1	225	346	661	1192	0
85	September 26, 2024				0	185	194	1016	1459	1
86	September 27, 2024			1	0	249	179	776	1078	0
87	September 27, 2024				0	123	232	682	1298	5
88	October 2, 2024			2	2	384	210	693	1109	0
89	October 2, 2024			1	1	206	307	743	1316	1
90	October 3, 2024			1	1	266	189	575	1094	0
91	October 3, 2024			1	1	330	140	723	1308	0
92	October 3, 2024				0	445	87	769	1191	0
93	October 3, 2024				0	335	147	911	1272	1
94	October 4, 2024				0	214	281	706	1157	0
95	October 4, 2024			1	1	170	192	827	1215	6
96	October 10, 2024			1	1	196	412	1103	1785	19
97	October 29, 2024			3	3	270	193	814	1360	0
98	October 29, 2024			1	1	353	319	792	1354	2
99	October 31, 2024				0	243	102	718	999	1
100	November 1, 2024				0	179	230	723	1184	6
101	November 7, 2024				0	231	230	737	1149	1
102	November 8, 2024			1	1	182	328	565	1130	14
103	November 22, 2024			1	1	237	297	760	1257	0
104	November 22, 2024				0	200	85	771	1136	0
105	November 22, 2024				0	280	249	832	1360	11
106	December 3, 2024			2	2	279	57	784	1110	0
107	December 3, 2024			1	2	269	224	988	1368	0
108	December 3, 2024			1	2	156	111	858	1153	0
109	December 3, 2024			1	2	184	145	678	1036	0
110	December 3, 2024				0	213	211	783	1150	2
111	December 5, 2024				0	156	235	805	1301	1
112	December 6, 2024			1	1	261	284	878	1346	1
113	December 7, 2024			1	1	184	205	797	1232	0
114	December 7, 2024				0	230	292	730	1205	0
115	December 7, 2024				0	183	192	728	1203	0
116	December 7, 2024			1	1	283	302	525	1049	4
117	December 11, 2024				5	265	279	713	1257	
118	December 11, 2024				0	222	276	661	1235	1
119	December 12, 2024				0	298	208	922	1403	1
120	December 13, 2024			1	1	273	196	597	1043	0
121	December 13, 2024				0	250	282	718	1424	22
122	January 4, 2025			1	1	424	179	651	1016	1
123	January 5, 2025				0	186	241	1101	1585	2
124	January 7, 2025				0	243	315	909	1454	0
125	January 7, 2025			1	1	230	206	672	1169	0
126	January 7, 2025			1	1	291	282	674	1173	1
127	January 8, 2025			1	1	217	157	653	1062	0
128	January 8, 2025				0	252	234	601	1192	2
129	January 10, 2025				0	357	352	787	1265	0
130	January 10, 2025			1	1	126	73	645	1199	1
131	January 11, 2025	1		1	2	481	133	622	970	0
132	January 11, 2025				0	215	138	646	1355	0
133	January 11, 2025				0	571	61	795	1085	0
134	January 11, 2025				0	229	306	853	1408	15
135	January 26, 2025				0	249	318	740	1298	0
136	January 26, 2025				0	240	203	587	1018	0
137	January 26, 2025				0	228	321	765	1264	0
138	January 26, 2025			2	2	178	247	574	999	0
139	January 26, 2025			1	1	178	252	720	1322	4
140	January 30, 2025				0	350	158	671	1066	0
141	January 30, 2025				0	237	268	888	1478	1
142	January 31, 2025				0	322	217	664	1068	0
143	January 31, 2025				0	187	96	619	943	0
144	January 31, 2025			1	1	228	377	1210	1932	28
145	February 28, 2025			1	1	345	269	670	1286	0
146	February 28, 2025			1	1	347	263	842	1527	1
147	March 1, 2025			1	1	422	213	940	1565	7
148	March 8, 2025				2	412	185	640	1051	0
149	March 8, 2025				2	226	314	862	1358	0
150	March 8, 2025				1	182	190	1224	1764	0
151	March 8, 2025				1	350	151	858	1248	1
152	March 9, 2025				1	239	140	625	1050	0
153	March 9, 2025				0	285	181	899	1343	10
154	March 19, 2025			1	1	263	290	861	1364	0
155	March 19, 2025			1	1	213	223	837	1333	1
156	March 20, 2025			1	1	273	244	738	1197	0
157	March 20, 2025			1	1	215	187	643	998	0
158	March 20, 2025				0	168	187	643	998	0

## References

[1] Andersen SA, Nayahangan LJ, Park YS, Konge L (2021). Use of generalizability theory for exploring reliability of and sources of variance in assessment of technical skills: a systematic review and meta-analysis. Acad Med.

[2] Asif H, McInnis C, Dang F (2022). Objective structured assessment of technical skill (OSATs) in the surgical skills and technology elective program (SSTEP): comparison of peer and expert raters. Am J Surg.

[3] M N, Sharma R, Suri A (2022). Microsurgical suturing assessment scores: a systematic review. Neurosurg Rev.

[4] Sugiyama T, Lama S, Gan LS (2018). Forces of tool-tissue interaction to assess surgical skill level. JAMA Surg.

[5] Sugiyama T, Sugimori H, Tang M (2024). Deep learning-based video-analysis of instrument motion in microvascular anastomosis training. Acta Neurochir (Wien).

[6] Tang M, Sugiyama T, Takahari R (2024). Assessment of changes in vessel area during needle manipulation in microvascular anastomosis using a deep learning-based semantic segmentation algorithm: a pilot study. Neurosurg Rev.

[7] Lavanchy JL, Zindel J, Kirtac K, Twick I, Hosgor E, Candinas D, Beldi G (2021). Author correction: automation of surgical skill assessment using a three-stage machine learning algorithm. Sci Rep.

[8] Soangra R, Sivakumar R, Anirudh ER, Reddy Y SV, John EB (2022). Evaluation of surgical skill using machine learning with optimal wearable sensor locations. PLoS One.

[9] Sugiyama T, Ito M, Sugimori H (2023). Tissue acceleration as a novel metric for surgical performance during carotid endarterectomy. Oper Neurosurg.

[10] Villanueva PJ, Sugiyama T, Villanueva BM, Rodriguez HI, Arciénaga A, Cherian I (2024). Using engineering methods (Kaizen and micromovements science) to improve and provide evidence regarding microsurgical hand skills. World Neurosurg.

[11] Villanueva PJ, Rodriguez HI, Sugiyama T, O'Keeffe D, Villanueva G, Villanueva BM, Roche AF (2025). Modeling the microsurgical learning curve using a Poisson-based statistical approach for skill assessment. Cureus.

[12] Villanueva P, Villanueva B, Arcienaga A, Rodriguez H, Lausada N, Lagier M (2024). A novel scoring system to reduce bias in placental microsurgery training. Seven Editora.

[13] Villanueva P, Villanueva BM, Sanmarco C (2023). Kaizen: engineering tools for development, evaluation, certification and continuous improvement of microsurgical abilities and procedures. Seven Editora.

[14] Imai K (1986). Kaizen (Ky'zen), The Key to Japan's Competitive Success. https://archive.org/details/kaizen00masa.

[15] Lizarelli FL, Antony J, Suarez M (2025). Analysis of the impact of Kaizen practices on ESG performance and the mediating role of digital systems. Business Process Management Journal.

[16] Datta V, Mackay S, Darzi A, Gillies D (2001). Motion analysis in the assessment of surgical skill. Comput Methods Biomech Biomed Engin.

[17] Ghasemloonia A, Maddahi Y, Zareinia K, Lama S, Dort JC, Sutherland GR (2017). Surgical skill assessment using motion quality and smoothness. J Surg Educ.

[18] Azari DP, Frasier LL, Quamme SR, Greenberg CC, Pugh CM, Greenberg JA, Radwin RG (2019). Modeling surgical technical skill using expert assessment for automated computer rating. Ann Surg.

